# Hospitalisations and Deaths Averted by COVID-19 Vaccination in Navarre, Spain, 2021–2022

**DOI:** 10.3390/vaccines12010058

**Published:** 2024-01-07

**Authors:** Iván Martínez-Baz, Camino Trobajo-Sanmartín, Ana Miqueleiz, Nerea Egüés, Manuel García Cenoz, Itziar Casado, Ana Navascués, Miguel Fernández-Huerta, Aitziber Echeverría, Marcela Guevara, Carmen Ezpeleta, Jesús Castilla

**Affiliations:** 1 Instituto de Salud Pública de Navarra, 31003 Pamplona, Spain; imartinba@navarra.es (I.M.-B.); camino.trobajo.sanmartin@navarra.es (C.T.-S.); mgcenoz@navarra.es (M.G.C.);; 2 CIBER Epidemiología y Salud Pública (CIBERESP), Instituto de Salud Carlos III, 28029 Madrid, Spain; 3 Instituto de Investigación Sanitaria de Navarra (IdiSNA), 31008 Pamplona, Spain; 4 Clinical Microbiology Department, Hospital Universitario de Navarra, 31008 Pamplona, Spain

**Keywords:** SARS-CoV-2, COVID-19 hospitalisation, COVID-19 mortality, COVID-19 vaccination, vaccination impact, vaccination effectiveness

## Abstract

In 2021–2022, most of the Spanish population received COVID-19 vaccines and a high proportion of them had SARS-CoV-2 infection. We estimated the rate of hospitalisations and deaths that were averted by risk reduction among vaccinated COVID-19 cases. Hospitalisations and deaths were analysed among COVID-19 cases confirmed in 2021 and 2022 in Navarre, Spain. To calculate the number of prevented outcomes by sex, age, comorbidities, and semester, the difference in the risk of each outcome between unvaccinated and vaccinated cases was multiplied by the number of vaccinated cases. COVID-19 vaccination coverage with any dose reached 88%, 86% with full vaccination, and 56% with a booster dose. The cumulative rates per 1000 inhabitants were 382 COVID-19 confirmed cases, 6.70 hospitalisations, and 1.15 deaths from COVID-19. The estimated rates of prevented events by vaccination were 16.33 hospitalisations and 3.39 deaths per 1000 inhabitants, which was 70.9% and 74.7% of expected events without vaccination, respectively. People aged 80 years and older or with major chronic conditions accounted for the majority of hospitalizations and deaths prevented by COVID-19 vaccination. One hospitalisation and death due to COVID-19 were averted for every 53 and 258 people vaccinated, respectively. The high COVID-19 vaccine effect in reducing the risk of severe outcomes and the high vaccination coverage in risk populations prevented three out of four hospitalisations and deaths due to COVID-19 during a period of intense circulation of SARS-CoV-2.

## 1. Introduction

Since the detection of the first case of COVID-19 in February 2020, SARS-CoV-2 circulated continuously in Spain for more than 3 years. The first wave of the COVID-19 pandemic accumulated a considerable number of hospitalisations and deaths due to COVID-19 in a few weeks, demonstrating the enormous pandemic capacity and severity of SARS-CoV-2 [1]. Non-pharmacological preventive measures were applied to modulate the circulation of SARS-CoV-2, but the relaxation in their application was usually followed by increases in infections, hospitalisations, and deaths [2,3].

In Spain, the COVID-19 vaccination campaign started on 27 December 2020 and was successively targeted to the population over 5 years old, from older to younger age groups. Immunocompromised people were prioritized regardless of age. Messenger RNA vaccines, Comirnaty (BNT162b2 mRNA, BioNTech-Pfizer, New York, United States) or Spikevax (mRNA-1273, Moderna, Cambridge, United States), were used for the majority of primary courses (~84%) and all booster doses, and adenovirus-based vaccines, Vaxzevria (ChAdOx1 nCoV-19, Oxford-AstraZeneca, Cambridge, United Kingdom) or Janssen vaccine (Ad26.COV2-S, Janssen-Cilag International NV, Beerse, Belgium) were used in all other primary courses [4,5]. 

High effectiveness of COVID-19 vaccination was reported in preventing hospitalisations and deaths due to COVID-19 (usually >80%), but effectiveness in preventing SARS-CoV-2 infection was lower and decreased over time [6,7,8,9,10,11,12,13,14,15,16,17,18,19,20]. This means that COVID-19 cases had a lower risk of hospitalisation and severe diseases if they were previously vaccinated [10]. Since COVID-19 vaccination was not able to control SARS-CoV-2 transmission over time, especially in the Omicron circulation period [20,21,22,23], the majority of the population was finally infected [24]. Therefore, the risk reduction in hospitalisation and death among vaccinated COVID-19 cases was responsible for most of the impact of COVID-19 vaccination on the health of the population [10]. 

Many studies have evaluated the COVID-19 vaccine effectiveness [6,7,8,9,10,11,12,13,14,15,16,17,18,19,20]; however, the whole impact of the COVID-19 vaccination programs during the pandemic has barely been evaluated, although these interventions were great challenges for public health. The number of events averted in the population is the main endpoint of a vaccination program and depends on the incidence of the event in unvaccinated people, the effectiveness of the vaccine, and the vaccination coverage, especially in high-risk groups [25]. This evaluation is essential to compare and prioritize among different public health interventions.

The seroprevalence of antinucleocapsid antibodies in the Navarre region, Spain, was 11% in November 2020 and increased to 62% in May 2022 [26,27], suggesting that more than half of the population was infected by SARS-CoV-2 during this period. The objective of this study was to estimate the number and rate of hospitalisations, intensive care unit (ICU) admissions, and deaths that were averted by risk reduction among vaccinated COVID-19 cases during a period of intense SARS-CoV-2 circulation.

## 2. Materials and Methods

### 2.1. Study Setting, Data Sources, and Design

This observational population-based study analysed information from the enhanced epidemiological surveillance of COVID-19 and the vaccination register in the region of Navarre, Spain (~660,000 inhabitants) [3], where the Regional Health Service provides medical care to residents free of charge at the point of use. This study included all people who tested positive for SARS-CoV-2 for the first time between January 2021 and December 2022. 

The enhanced surveillance of COVID-19 was based on the electronic reporting of all confirmed cases from public and private healthcare centres and laboratories, including diagnoses using the polymerase chain reaction (PCR) and antigen tests on nasopharyngeal swabs. Diagnoses performed in pharmacies and those reported by the patient through the established online procedure were also included. 

The COVID-19 outcomes evaluated were hospitalisation, ICU admission, and death. Within the epidemiological surveillance, medical doctors reviewed the outcomes that occurred during the 3 months after the SARS-CoV-2 positive test. COVID-19 hospitalised cases were defined as patients admitted for 24 h or more or who died due to COVID-19. Deaths due to confirmed COVID-19 that occurred outside the hospital were also assessed. 

### 2.2. COVID-19 Vaccination and Covariables

The regional vaccination register was used to obtain the number of doses of COVID-19 vaccine, date of immunisation, and product. A case was considered ‘fully vaccinated’ after receiving 1 dose of the Janssen vaccine or 2 doses of other products, and ‘partially vaccinated’ if only 1 dose of these other products was received. Booster doses after the primary series were also considered. Each vaccine dose was counted 14 days after administration. 

Vaccination status was considered in the following 7 categories: (1) unvaccinated (reference category); (2) partially vaccinated; (3) fully vaccinated in the first 6 months before the positive test; (4) fully vaccinated more than 6 months before the positive test; (5) a booster dose in the first 6 months before the positive test; (6) a booster dose more than 6 months before the positive test; and (7) a second booster dose. 

Study variables included sex, age, major chronic conditions, COVID-19 vaccination status, and date of COVID-19 diagnosis. Diagnoses of major chronic conditions were obtained from primary healthcare electronic medical records and included immune compromise and other chronic conditions (cardiovascular diseases, diabetes mellitus, liver cirrhosis, chronic kidney disease, chronic obstructive pulmonary diseases, asthma, dementia, stroke, rheumatic diseases, cancer, and body mass index  ≥  40 kg/m^2^).

Information on SARS-CoV-2 test results (PCR, antigen test, and self-testing), vaccination status, and other variables related to the same individual were linked using a unique personal identifier.

Four semesters were considered in the study period. The period from January to June 2021 was characterised by a progressive increase in vaccination coverage against COVID-19 and the circulation of the EU1 lineage and the Alpha variant. From July to December 2021, children’s vaccination started and elderly adults received the first booster dose, while the Delta variant became dominant. January to June 2022 was characterised by high vaccination coverage, a progressive extension of a booster dose to adults, and predominance of circulation of the Omicron BA.1 and BA.2 subvariants. July to December 2022 was characterised by very high coverage of full vaccination or with a booster dose and progressive administration of a second booster dose in the high-risk population, while the Omicron BA.4 and BA.5 subvariants circulated [28].

### 2.3. Statistical Analysis

A counterfactual evaluation compared the observed COVID-19 outcomes with those that would have occurred in the same population, during the same period, and under the same conditions, assuming that no COVID-19 cases had been previously vaccinated against COVID-19.

Among COVID-19 confirmed cases, the risk of each outcome (hospitalisation, ICU admission, and death) was calculated by vaccination status categories, sex, age groups (0–4, 5–19, 20–34, 35–49, 50–64, 65–79, and ≥ 80 years), underlying conditions (immunocompromised, other underlying chronic conditions, and none), and semester. The number of prevented outcomes by risk reduction among vaccinated COVID-19 cases was calculated as the risk difference between unvaccinated individuals and those in each vaccination category multiplied by the number of COVID-19 cases in the respective vaccination status category. This analysis was stratified for each category combining sex, age group, presence of underlying condition, and semester. The total impact of vaccination was the sum of the number of events prevented in each category. The estimated number of events prevented was used to calculate the rate per 1000 inhabitants and the percentage they represented among the total events expected in the absence of vaccination, including observed and prevented events. The annual population covered by the Navarre Health Service was used to calculate vaccination coverage and incidence rates by sex, age, and major chronic conditions.

For the entire study period, the number of people vaccinated for each event prevented by the reduced risk among vaccinated COVID-19 cases was calculated by dividing the number of people vaccinated by the number of events prevented.

## 3. Results

### 3.1. COVID-19 Vaccination Coverage and Incidence of COVID-19 Outcomes 

**Figure 1:**
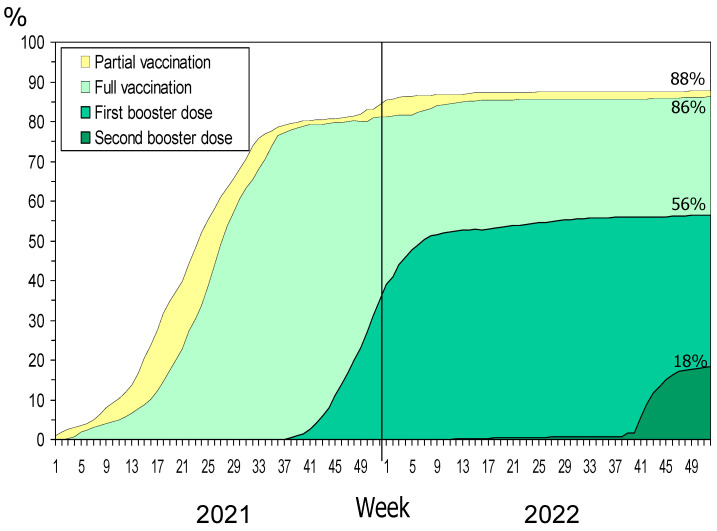
COVID-19 vaccination status per week in the general population.

At the end of 2021, 84% of the population had received any dose of COVID-19 vaccine, 81% were fully vaccinated, and 35% had received one booster dose. In December 2022, the coverage increased to 88%, 86% and 56%, respectively, and 18% had received a second booster dose (Figure 1).

In the 2021–2022 period, 252,108 persons were confirmed with COVID-19 for the first time (382 per 1000 inhabitants), 4419 of them were hospitalised due to COVID-19 (6.70 per 1000 inhabitants), 447 were admitted to ICU (0.68 per 1000), and 756 died from COVID-19 (1.15 per 1000 inhabitants). More than half of the cases were diagnosed in the first semester of 2022. 

Of COVID-19 cases confirmed for the first time during the study period, 73.0% had received any COVID-19 vaccine dose, 69.0% were fully vaccinated, and 29.8% had received any booster dose. The proportion of COVID-19 cases who had received any dose of the COVID-19 vaccine increased from 4.3% in the first semester of 2021 to 93.3% in the second semester of 2022. Vaccination coverage was higher in women, older age groups, and immunocompromised people (Table 1).

### 3.2. Hospitalisations and Deaths Averted by COVID-19 Vaccination

During 2021 and 2022, it was estimated that 10,767 hospitalisations, 1375 ICU admissions, and 2232 deaths due to COVID-19 were averted because COVID-19 cases had previously received any COVID-19 vaccine dose. Averted events comprised 16.33 hospitalisations, 2.09 ICU admissions, and 3.39 deaths per 1000 inhabitants, and 70.9%, 75.5%, and 74.7% of the events that would have been expected to occur in the absence of vaccination, respectively. 

The rates of observed and prevented hospitalisations were higher in men than in women, but the rate of averted deaths was higher in women. The rates of prevented hospitalisations and deaths progressively increased with age and were higher in immunocompromised patients. Patients with major chronic conditions without immune compromise represented 77% of hospitalizations, 66% of ICU admissions, and 68% of deaths prevented by COVID-19 vaccination (Table 2, Table 3 and Table 4).

The percentage of expected hospitalisations that were prevented by COVID-19 vaccination was higher than 70% in people older than 50 years and in patients with major chronic conditions without immune compromise. On average, this percentage increased until the first semester of 2022 (83.4%) and declined in the second semester of 2022 (62.5%). 

The rates of observed COVID-19 hospitalisations and those prevented by COVID-19 vaccination were much higher in people aged 50 years and older than in the younger population (Table 2). The highest rates of ICU admission, both observed and prevented, occurred in people aged 50 to 79 years old (Table 3). Most of the observed deaths due to COVID-19 (72%) and those averted by COVID-19 vaccination (78%) were concentrated in the population aged 80 years and older (Table 4). 

### 3.3. Impact of COVID-19 Vaccination over Time

In the first semester of 2021, COVID-19 vaccination coverage in the population grew rapidly up to 58%. Risk reduction in previously vaccinated COVID-19 cases averted 5.8%, 5.1%, and 6.0% of all expected hospitalisations, ICU admissions, and deaths due to COVID-19 in the population, respectively. In the second semester of 2021, the vaccination coverage increased to 84%, and the proportion of COVID-19 outcomes averted by risk reduction among vaccinated COVID-19 cases increased to 75.4% of expected hospitalisations, 82.3% of ICU admissions, and 71.7% of deaths. The highest incidence of COVID-19 cases was observed in the first semester of 2022, when the percentages of averted outcomes increased to 83.4%, 90.0%, and 85.2%, respectively. In the second semester of 2022, the incidence of COVID-19 cases and all analysed outcomes decreased, and the proportion of averted events also decreased to 62.5%, 57.0%, and 54.0%, respectively (Table 2, Table 3 and Table 4). 

**Figure 2:**
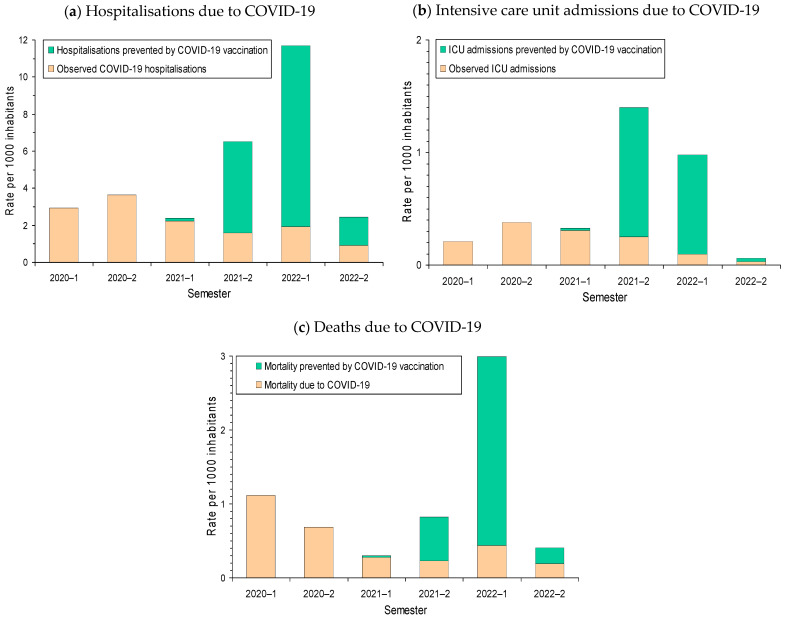
Biannual rate of COVID-19 outcomes per 1000 inhabitants and estimated rate of outcomes averted by risk reduction in vaccinated COVID-19 cases: (**a**) Hospitalisation; (**b**) intensive care unit admission (ICU); and (**c**) death due to COVID-19. The estimated number of events prevented was obtained as the sum of the results from analyses stratified for each category combining sex, age group, presence of major chronic condition, semester, and COVID-19 vaccination status.

The highest rates of COVID-19 hospitalisations were observed in 2020 before the availability of COVID-19 vaccines. The proportion of expected hospitalisations that was averted by risk reduction in vaccinated COVID-19 cases gradually increased during 2021 and, in the second semester of 2021, it exceeded half of the expected hospitalisations. In the absence of vaccination, the rate of hospitalizations in the first semester of 2022 would have multiplied by three or four the rate of hospitalisations observed in the pre-vaccination periods. The population impact of COVID-19 vaccination in reducing the risk of ICU admission among vaccinated COVID-19 cases was more pronounced in the second semester of 2021, while the impact in reducing the risk of death due to COVID-19 was especially pronounced in the first semester of 2022 (Figure 2).

### 3.4. Vaccine Doses Administered and People Vaccinated for Each Event Prevented

On average, during the study period, we estimated that for every 134, 1046, and 644 doses of vaccine administered, one hospitalisation, one ICU admission, and one death due to COVID-19 were averted, respectively, by risk reduction in vaccinated COVID-19 cases. One hospitalisation was averted for every 53 COVID-19 vaccinated persons, one ICU admission for every 419, and one death due to COVID-19 for every 258 vaccinated persons.

## 4. Discussion

The present study estimates that COVID-19 vaccination avoided a considerable number and proportion of the expected hospitalisations, ICU admissions, and deaths among COVID-19 cases in Navarre. This impact markedly softened the dramatic scenario that would be expected in the absence of COVID-19 vaccination. This considerable impact in the prevention of hospitalisations and deaths was due to the coincidence of several circumstances: the extremely high incidence of SARS-CoV-2 infections, especially since the spread of the Omicron variant [3,26]; the high effectiveness of the vaccine in risk reduction in severe outcomes among COVID-19 cases regardless of the SARS-CoV-2 variant [6,7,8,9,10,11,12,13,14,15,16,17,18]; the large full vaccination coverage that was quickly achieved in the population, firstly, with the complete vaccination schedule, and latterly, with a booster dose in the high-risk people; and the appropriate prioritisation of vaccination to people at highest risk of developing severe infection outcomes [4]. The confluence of all these circumstances led to the success of this vaccination program. 

On average, only 53 people vaccinated were sufficient to prevent one COVID-19 hospitalisation and 258 people vaccinated to prevent one death due to COVID-19. These findings show the high efficiency of this vaccination program in the context of the COVID-19 pandemic.

COVID-19 vaccination initially showed an important preventive effect of infections and a powerful risk reduction in severe forms of the disease [6,7]. The vaccination effectiveness in preventing transmission decreased over time since the last dose and was also lower against the successive variants of concern, Alpha, Delta, and Omicron [8,23]. In addition, the relaxation in adherence to non-pharmacological preventive measures among vaccinated people could counteract part of the vaccine effect in preventing infections [29]. 

By the end of the study period, the majority of the population had received a SARS-CoV-2 infection [30], which demonstrated that the risk reduction in severe outcomes among vaccinated COVID-19 cases was more relevant for the vaccination impact than the effect in preventing SARS-CoV-2 infection. 

The proportion of severe outcomes that were prevented increased rapidly with the rising coverage of full vaccination and booster doses in the high-risk population. Although COVID-19 cases were more frequently vaccinated in women than men, the rates of observed and averted hospitalisations were higher in men, suggesting greater vulnerability and potential benefit of vaccination for men. Women had a higher impact of COVID-19 vaccination in preventing deaths, probably due to the more complete vaccination coverage. The vaccination impact was earlier and more intense in the elderly because they had a higher risk of hospitalisation and death in the case of being infected and because they were offered each dose of vaccine first [4]. Most of the deaths prevented by COVID-19 vaccination (78%) were concentrated in the population aged 80 years and older.

Although the rates of prevented hospitalisations and deaths were higher in immunocompromised patients, those with major chronic conditions without immune compromise accounted for the majority of hospitalizations, ICU admissions, and deaths prevented by COVID-19 vaccination because they were a large population group and accounted for most of the serious outcomes of the SARS-CoV-2 infection [31].

The greatest impact of vaccination on the number of hospitalisations and deaths averted was observed in the first semester of 2022, given the much higher incidence of infections during the Omicron period, the relaxation of non-pharmacological preventive measures [2,3], and the high vaccination coverage in the high-risk population. As a result, the percentages of the expected hospitalisations, ICU admissions, and deaths due to COVID-19, which were averted in the first semester of 2022, rose to 83.4%, 90.0%, and 85.2%, respectively. The results show that in the absence of vaccination, the rates of hospitalisations and deaths due to COVID-19 during the Omicron period would have far exceeded that observed in the periods before the vaccine was available. Although the percentage of ICU admissions averted was higher in the first semester of 2022, the number of ICU admissions averted was higher in the second semester of 2021, which can be explained by the greater tendency of the Delta variant than Omicron to cause severe outcomes [32,33].

To the best of our knowledge, only one study in the Netherlands estimated the number of hospitalisations averted by COVID-19 vaccination during the period when most of the population had received their first COVID-19 infection [34]. Other studies evaluated the impact of COVID-19 vaccination only during the first year of the vaccination campaign [34,35,36,37,38]. These studies estimated that 56–74% of hospitalisations and 58–82% of deaths were averted by COVID-19 vaccination [34,35,36,37,38], which is consistent with the results of our study at that time.

The risk reduction and the proportion of expected COVID-19 outcomes that were averted by COVID-19 vaccination declined in the second semester of 2022. By then, a high proportion of the population had been infected by SARS-CoV-2 regardless of their vaccination status, although many of these infections had not been confirmed [3,30]. These infections produce some natural protection that may dilute the estimates of COVID-19 vaccination effect and impact [30]. 

In interpreting this study, some aspects must be taken into account. The estimates came from a counterfactual analysis, which compared the observed reality with a theoretical scenario of no vaccination against COVID-19 but maintained all the other conditions unchanged. However, it seems unlikely that such steep increases in the incidence of severe COVID-19 outcomes would not have prompted additional non-pharmacological preventive measures. Therefore, what vaccination possibly contributed to was not avoiding all these events, but rather allowing the normalisation of productive activity and social life, while maintaining a moderate and manageable incidence of serious illness and deaths due to COVID-19.

The vaccine effect to prevent SARS-CoV-2 infections was not considered; therefore, the total impact of vaccination may be even greater. However, since COVID-19 vaccines have not achieved complete control of SARS-CoV-2 transmission [27,28] but have achieved a high effect in reducing the risk of hospitalisation and death due to COVID-19, the main impact of vaccination is expected as a result of this effect [10].

This study only considered the first COVID-19 diagnosis of each person. Since hospitalisations and deaths due to COVID-19 reinfections were not considered, the total number of events prevented would be even higher. However, the risk of a second infection and its severity tends to be much lower than that of the first one, so the first episodes capture most of the severe disease burden [39,40]. 

The present study has the advantage of a case-to-case comparison. COVID-19 cases were obtained from the same information source regardless of vaccination status, which ensures the comparability of data. The calculations were made in homogeneous strata with respect to the expected effect of vaccination because the analyses were stratified by the combinations of sex, age group, comorbidity, semester, and vaccination status. 

Although children younger than 5 years were excluded from COVID-19 vaccination, they were included in the analysis to achieve population-based results. The rate and proportion of averted events will be higher in the analysis restricted to the target population for COVID-19 vaccination. 

The present study has some limitations. An undiagnosed first infection was probably more common among unvaccinated people [30], which could lead to an underestimation of the effectiveness and impact of vaccination. Variability in the COVID-19 vaccination effect in reducing the risk of severe outcomes by vaccine product and variant was not considered [8,33], but the stratification by age and semester provided a good control of these effects because changes in circulating variants roughly coincided with semester changes [3]. This study was performed in a region where the large majority of the population had the first COVID-19 infection after being fully vaccinated; therefore, the results may differ from other sites where people were mainly infected before vaccination. The epidemiological and vaccination conditions of this study may not be similar to other sites.

## 5. Conclusions

In conclusion, vaccination against COVID-19 in Navarre had a very relevant impact on the prevention of hospitalisations, ICU admissions, and deaths due to COVID-19 since it completely changed the severity and lethality of this disease. This impact was the result of the high vaccine effect on risk reduction in severe outcomes among COVID-19 cases, the high vaccination coverage, and the prioritisation of vaccination in the high-risk population in a period of intense SARS-CoV-2 circulation. The confluence of all these circumstances led to the spectacular success of this vaccination program. The COVID-19 vaccination campaign entailed an extraordinary scientific, organisational, and economic effort that had a considerable impact on the population’s health. In the absence of COVID-19 vaccination, the progressive normalisation of the COVID-19 infection that took place in Europe in 2022 would have produced a significant number of additional hospitalisations and deaths. Fortunately, most hospitalisations and deaths were avoided owing to the COVID-19 vaccination. Rapid vaccine development may be essential to minimise the impact of pandemics.

## Figures and Tables

**Table 1:** COVID-19 confirmed cases and COVID-19 vaccination status at COVID-19 diagnosis.

	COVID-19 Confirmed Cases in 2021–2022		COVID-19 Vaccination Status at COVID-19 Diagnosis %			
	Number	Rate per 1000 Inhabitants	Any Dose	Full Vaccination	First Booster Dose	Second Booster Dose
**Sex**						
Men	116,945	359	70.3	66.0	25.9	0.7
Women	135,163	405	75.3	71.6	33.1	0.8
**Age, years**						
0–4	9734	352	0	0	0	0
5–19	44,176	415	43.8	32.6	0.4	0
20–34	43,459	416	68.9	66.3	11.5	0.1
35–49	61,210	424	83.8	80.8	22.5	0.3
50–64	47,905	339	86.6	83.3	41.6	0.9
65–79	30,619	328	91.6	90.2	76.8	2.5
80+	15,005	358	92.9	91.6	84.5	3.0
**Major chronic condition**						
Immunocompromised	3862	358	85.3	83.3	55.0	8.9
Other chronic condition	73,686	358	81.9	78.6	46.1	1.3
No	174,560	394	69.0	64.6	22.3	0.3
**Semester**						
2021–1	22,264	34	4.3	1.0	0	0
2021–2	74,568	113	64.7	60.4	4.4	0
2022–1	130,260	198	85.6	81.0	39.9	0.3
2022–2	25,016	38	93.3	92.8	79.3	6.0
**Total**	252,108	382	73.0	69.0	29.8	0.7

**Table 2:** Observed number and rate of hospitalizations due to COVID-19 and estimated hospitalizations prevented by risk reduction in vaccinated COVID-19 cases.

	Observed Hospitalisations		Estimated Hospitalisations Prevented ^a^		
	Number	Rate per 1000 Inhabitants	Number	Rate per 1000 Inhabitants	Prevented Events among the Total Expected in the Absence of Vaccination, %
**Sex**					
Men	2469	7.58	5633	17.31	69.5
Women	1950	5.84	5134	15.39	72.5
**Age, years**					
0–4	97	3.51	0	0	0
5–19	34	0.32	5	0.05	12.7
20–34	160	1.53	143	1.37	47.2
35–49	475	3.29	915	6.34	65.8
50–64	932	6.60	2256	15.98	70.8
65–79	1211	12.99	3439	36.87	74.0
80+	1510	36.07	4010	95.80	72.6
**Major chronic condition**					
Immunocompromised	319	29.57	500	46.37	61.1
Other chronic condition	2665	12.94	7338	35.65	73.4
No	1435	3.24	2929	6.62	67.1
**Semester**					
2021–1	1480	2.25	92	0.14	5.8
2021–2	1053	1.60	3234	4.91	75.4
2022–1	1277	1.94	6429	9.75	83.4
2022–2	609	0.92	1013	1.54	62.5
**Total**	4419	6.70	10,767	16.33	70.9

^a^ The estimated number of hospitalisations prevented was obtained as the sum of the results from analyses stratified for each category combining sex, age group, presence of major chronic condition, semester, and COVID-19 vaccination status.

**Table 3:** Observed number and rate of intensive care unit (ICU) admissions due to COVID-19 and prevented admissions by risk reduction in vaccinated COVID-19 cases.

	Observed ICU Admissions		Estimated ICU Admissions Prevented ^a^		
	Number	Rate per 1000 Inhabitants	Number	Rate per 1000 Inhabitants	Prevented Events among the Total Expected in the Absence of Vaccination, %
**Sex**					
Men	289	0.89	770	2.37	72.7
Women	158	0.47	604	1.81	79.3
**Age, years**					
0–4	4	0.14	0	0	0
5–19	2	0.02	0	0	0
20–34	23	0.22	23	0.22	49.7
35–49	64	0.44	184	1.27	74.2
50–64	157	1.11	640	4.54	80.3
65–79	182	1.95	468	5.02	72.0
80+	15	0.36	60	1.44	80.1
**Major chronic condition**					
Immunocompromised	36	3.34	17	1.58	32.1
Other chronic condition	247	1.20	914	4.44	78.7
No	164	0.37	444	1.00	73.0
**Semester**					
2021–1	202	0.31	11	0.02	5.1
2021–2	163	0.25	758	1.15	82.3
2022–1	65	0.10	583	0.88	90.0
2022–2	17	0.03	23	0.03	57.0
**Total**	447	0.68	1375	2.09	75.5

^a^ The estimated number of intensive care unit admissions prevented was obtained as the sum of the results from analyses stratified for each category combining sex, age group, presence of major chronic condition, semester, and COVID-19 vaccination status.

**Table 4:** Observed number and rate of deaths due to COVID-19 and prevented deaths by risk reduction in vaccinated COVID-19 cases.

	Observed Deaths		Estimated Deaths Prevented ^a^		
	Number	Rate per 1000 Inhabitants	Number	Rate per 1000 Inhabitants	Prevented Events among the Total Expected in the Absence of Vaccination, %
**Sex**					
Men	411	1.26	1079	3.31	72.4
Women	345	1.03	1154	3.46	77.0
**Age, years**					
0–4	0	0	0	0	0
5–19	0	0	0	0	0
20–34	1	0.01	0	0	0
35–49	4	0.03	5	0.04	56.8
50–64	40	0.28	83	0.59	67.4
65–79	166	1.78	414	4.44	71.4
80+	545	13.02	1730	41.33	76.0
**Major chronic condition**					
Immunocompromised	52	4.82	195	18.03	78.9
Other chronic condition	566	2.75	1712	8.32	75.2
No	138	0.31	326	0.74	70.2
**Semester**					
2021–1	184	0.28	12	0.02	6.0
2021–2	153	0.23	388	0.59	71.7
2022–1	293	0.44	1684	2.55	85.2
2022–2	126	0.19	148	0.22	54.0
**Total**	756	1.15	2232	3.39	74.7

^a^ The estimated number of deaths prevented was obtained as the sum of the results from analyses stratified for each category combining sex, age group, presence of major chronic condition, semester, and COVID-19 vaccination status.

## Data Availability

The data presented in this study are available upon request from the corresponding author.

## References

[1] Working Group for the Surveillance and Control of COVID-19 in Spain (2020). The first wave of the COVID-19 pandemic in Spain: Characterisation of cases and risk factors for severe outcomes, as at 27 April 2020. Eurosurveillance.

[2] García-García D., Herranz-Hernández R., Rojas-Benedicto A., León-Gómez I., Larrauri A., Peñuelas M., Guerrero-Vadillo M., Ramis R., Gómez-Barroso D. (2022). Assessing the effect of non-pharmaceutical interventions on COVID-19 transmission in Spain, 30 August 2020 to 31 January 2021. Eurosurveillance.

[3] Casado I., García Cenoz M., Egüés N., Burgui C., Martínez-Baz I., Castilla J. (2023). Infecciones, hospitalizaciones y mortalidad por COVID-19 en Navarra entre febrero de 2020 y septiembre de 2022 [COVID-19 infections, hospitalizations, and morality in Navarre (Spain) between February 2020 and September 2022]. An. Sist. Sanit. Navar..

[4] Grupo de Trabajo Técnico de Vacunación COVID-19, de la Ponencia de Programa y Registro de Vacunaciones Estrategia de vacunación frente a COVID-19 en España. https://www.sanidad.gob.es/profesionales/saludPublica/prevPromocion/vacunaciones/covid19/Actualizaciones_Estrategia_Vacunacion/docs/COVID-19_EstrategiaVacunacion.pdf.

[5] Ministerio de Sanidad (2021). GIV COVID-19. Gestión Integral de la Vacunación COVID-19. Date 30/11/2021 [Spanish Ministry of Health. Integrate Management of the COVID-19 Vaccination Programme. Report 30/11/2021].

[6] Thompson M.G., Stenehjem E., Grannis S., Ball S.W., Naleway A.L., Ong T.C., DeSilva M.B., Natarajan K., Bozio C.H., Lewis N. (2021). Effectiveness of COVID-19 Vaccines in Ambulatory and Inpatient Care Settings. N. Engl. J. Med..

[7] Martínez-Baz I., Miqueleiz A., Casado I., Navascués A., Trobajo-Sanmartín C., Burgui C., Guevara M., Ezpeleta C., Castilla J., Working Group for the Study of COVID-19 in Navarra (2021). Effectiveness of COVID-19 vaccines in preventing SARS-CoV-2 infection and hospitalisation, Navarre, Spain, January to April 2021. Eurosurveillance.

[8] de Gier B., Andeweg S., Joosten R., Ter Schegget R., Smorenburg N., van de Kassteele J., Hahné S.J., van den Hof S., de Melker H.E., RIVM COVID-19 Surveillance and Epidemiology Team (2021). Vaccine effectiveness against SARS-CoV-2 transmission and infections among household and other close contacts of confirmed cases, the Netherlands, February to May 2021. Eurosurveillance.

[9] Vasileiou E., Simpson C.R., Shi T., Kerr S., Agrawal U., Akbari A., Bedston S., Beggs J., Bradley D., Chuter A. (2021). Interim findings from first-dose mass COVID-19 vaccination roll-out and COVID-19 hospital admissions in Scotland: A national prospective cohort study. Lancet.

[10] Tenforde M.W., Olson S.M., Self W.H., Talbot H.K., Lindsell C.J., Steingrub J.S., Shapiro N.I., Ginde A.A., Douin D.J., Prekker M.E. (2021). Effectiveness of Pfizer-BioNTech and Moderna Vaccines Against COVID-19 Among Hospitalized Adults Aged ≥65 Years—United States, January-March 2021. MMWR Morb. Mortal. Wkly. Rep..

[11] Haas E.J., Angulo F.J., McLaughlin J.M., Anis E., Singer S.R., Khan F., Brooks N., Smaja M., Mircus G., Pan K. (2021). Impact and effectiveness of mRNA BNT162b2 vaccine against SARS-CoV-2 infections and COVID-19 cases, hospitalisations, and deaths following a nationwide vaccination campaign in Israel: An observational study using national surveillance data. Lancet.

[12] Martínez-Baz I., Trobajo-Sanmartín C., Miqueleiz A., Casado I., Navascués A., Burgui C., Ezpeleta C., Castilla J., Guevara M., Working Group for the Study of COVID-19 in Navarra (2023). Risk reduction of hospitalisation and severe disease in vaccinated COVID-19 cases during the SARS-CoV-2 variant Omicron BA.1-predominant period, Navarre, Spain, January to March 2022. Eurosurveillance.

[13] Andrews N., Stowe J., Kirsebom F., Toffa S., Rickeard T., Gallagher E., Gower C., Kall M., Groves N., O’Connell A.M. (2022). COVID-19 vaccine effectiveness against the Omicron (B.1.1.529) variant. N. Engl. J. Med..

[14] Hall V., Foulkes S., Insalata F., Kirwan P., Saei A., Atti A., Wellington E., Khawam J., Munro K., Cole M. (2022). Protection against SARS-CoV-2 after COVID-19 vaccination and previous infection. N. Engl. J. Med..

[15] Tartof S.Y., Slezak J.M., Fischer H., Hong V., Ackerson B.K., Ranasinghe O.N., Frankland T.B., Ogun O.A., Zamparo J.M., Gray S. (2021). Effectiveness of mRNA BNT162b2 COVID-19 vaccine up to 6 months in a large integrated health system in the USA: A retrospective cohort study. Lancet.

[16] Andrews N., Tessier E., Stowe J., Gower C., Kirsebom F., Simmons R., Gallagher E., Thelwall S., Groves N., Dabrera G. (2022). Duration of protection against mild and severe disease by COVID-19 vaccines. N. Engl. J. Med..

[17] Rose A.M., Nicolay N., Sandonis Martín V., Mazagatos C., Petrović G., Niessen F.A., Machado A., Launay O., Denayer S., Seyler L. (2023). Vaccine effectiveness against COVID-19 hospitalisation in adults (≥20 years) during Alpha- and Delta-dominant circulation: I-MOVE-COVID-19 and VEBIS SARI VE networks, Europe, 2021. Eurosurveillance.

[18] Rose A.M., Nicolay N., Sandonis Martín V., Mazagatos C., Petrović G., Baruch J., Denayer S., Seyler L., Domegan L., Launay O. (2023). Vaccine effectiveness against COVID-19 hospitalisation in adults (≥20 years) during Omicron-dominant circulation: I-MOVE-COVID-19 and VEBIS SARI VE networks, Europe, 2021 to 2022. Eurosurveillance.

[19] Feikin D.R., Higdon M.M., Abu-Raddad L.J., Andrews N., Araos R., Goldberg Y., Groome M.J., Huppert A., O’Brien K.L., Smith P.G. (2022). Duration of effectiveness of vaccines against SARS-CoV-2 infection and COVID-19 disease: Results of a systematic review and meta-regression. Lancet.

[20] Bobrovitz N., Ware H., Ma X., Li Z., Hosseini R., Cao C., Selemon A., Whelan M., Premji Z., Issa H. (2023). Protective effectiveness of previous SARS-CoV-2 infection and hybrid immunity against the omicron variant and severe disease: A systematic review and meta-regression. Lancet Infect. Dis..

[21] Lauring A.S., Tenforde M.W., Chappell J.D., Gaglani M., Ginde A.A., McNeal T., Ghamande S., Douin D.J., Talbot H.K., Casey J.D. (2022). Clinical severity of, and effectiveness of mRNA vaccines against, COVID-19 from omicron, delta, and alpha SARS-CoV-2 variants in the United States: Prospective observational study. BMJ.

[22] Eggink D., Andeweg S.P., Vennema H., van Maarseveen N., Vermaas K., Vlaemynck B., Schepers R., van Gageldonk-Lafeber A.B., van den Hof S., Reusken C.B. (2022). Increased risk of infection with SARS-CoV-2 Omicron BA.1 compared with Delta in vaccinated and previously infected individuals, the Netherlands, 22 November 2021 to 19 January 2022. Eurosurveillance.

[23] Nyberg T., Ferguson N.M., Nash S.G., Webster H.H., Flaxman S., Andrews N., Hinsley W., Bernal J.L., Kall M., Bhatt S. (2022). Comparative analysis of the risks of hospitalisation and death associated with SARS-CoV-2 Omicron (B.1.1.529) and Delta (B.1.617.2) variants in England: A cohort study. Lancet.

[24] European Centre for Disease Prevention and Control (2021). Implications of the Spread of the SARS-CoV-2 B.1.1.529 Variant of Concern (Omicron) for the EU/EEA—First Update. 2 December 2021.

[25] Halloran M.E., Struchiner C.J., Longini I.M. (1997). Study designs for evaluating different efficacy and effectiveness aspects of vaccines. Am. J. Epidemiol..

[26] Instituto de Salud Carlos III Estudio ENE-COVID: Cuarta Ronda. https://portalcne.isciii.es/enecovid19/informes/informe_cuarta_ronda.pdf.

[27] Health Department, Government of Navarre (2022). Encuesta de Seroprevalencia de Anticuerpos Frente al SARS-CoV-2 en Pacientes de Atención Primaria de Navarra. [Seroprevalence of Antibodies against SARS-CoV-2 in Navarre].

[28] Centro de Coordinación de Alertas y Emergencias Sanitarias (2023). Informe Sobre la Pandemia de COVID-19 en España. 30 de junio de 2023.

[29] Martínez-Baz I., Miqueleiz A., Egüés N., Casado I., Burgui C., Echeverría A., Navascués A., Fernández-Huerta M., García Cenoz M., Trobajo-Sanmartín C. (2023). Effect of COVID-19 vaccination on the SARS-CoV-2 transmission among social and household close contacts: A cohort study. J. Infect. Public Health.

[30] Castilla J., Lecea Ó., Martín Salas C., Quílez D., Miqueleiz A., Trobajo-Sanmartín C., Navascués A., Martínez-Baz I., Casado I., Burgui C. (2022). Seroprevalence of antibodies against SARS-CoV-2 and risk of COVID-19 in Navarre, Spain, May to July 2022. Eurosurveillance.

[31] de Lusignan S., Dorward J., Correa A., Jones N., Akinyemi O., Amirthalingam G., Andrews N., Byford R., Dabrera G., Elliot A. (2020). Risk factors for SARS-CoV-2 among patients in the Oxford Royal College of General Practitioners Research and Surveillance Centre primary care network: A cross-sectional study. Lancet Infect. Dis..

[32] Ulloa A.C., Buchan S.A., Daneman N., Brown K.A. (2022). Estimates of SARS-CoV-2 Omicron variant severity in Ontario, Canada. JAMA.

[33] Trobajo-Sanmartín C., Miqueleiz A., Guevara M., Fernández-Huerta M., Burgui C., Casado I., Baigorria F., Navascués A., Ezpeleta C., Castilla J. (2023). Comparison of the risk of hospitalization and severe disease among co-circulating severe acute respiratory syndrome coronavirus 2 variants. J. Infect. Dis..

[34] van Iersel S.C.J.L., McDonald S.A., de Gier B., Knol M.J., de Melker H.E., Henri van Werkhoven C.H., Hahné S.J.M., RIVM COVID-19 Epidemiology and Surveillance Team (2023). Number of COVID-19 hospitalisations averted by vaccination: Estimates for the Netherlands, January 6, 2021 through August 30, 2022. Vaccine.

[35] Sacco C., Mateo-Urdiales A., Petrone D., Spuri M., Fabiani M., Vescio M.F., Bressi M., Riccardo F., Del Manso M., Bella A. (2021). Estimating averted COVID-19 cases, hospitalisations, intensive care unit admissions and deaths by COVID-19 vaccination, Italy, January-September 2021. Eurosurveillance.

[36] Steele M.K., Couture A., Reed C., Iuliano D., Whitaker M., Fast H., Hall A.J., MacNeil A., Cadwell B., Marks K.J. (2022). Estimated Number of COVID-19 Infections, Hospitalizations, and Deaths Prevented Among Vaccinated Persons in the US, December 2020 to September 2021. JAMA Netw. Open.

[37] Kayano T., Sasanami M., Kobayashi T., Ko Y.K., Otani K., Suzuki M., Nishiura H. (2022). Number of averted COVID-19 cases and deaths attributable to reduced risk in vaccinated individuals in Japan. Lancet Reg. Health West. Pac..

[38] Meslé M.M., Brown J., Mook P., Hagan J., Pastore R., Bundle N., Spiteri G., Ravasi G., Nicolay N., Andrews N. (2021). Estimated number of deaths directly averted in people 60 years and older as a result of COVID-19 vaccination in the WHO European Region, December 2020 to November 2021. Eurosurveillance.

[39] Altarawneh H.N., Chemaitelly H., Ayoub H.H., Tang P., Hasan M.R., Yassine H.M., Al-Khatib H.A., Smatti M.K., Coyle P., Al-Kanaani Z. (2022). Effects of Previous Infection and Vaccination on Symptomatic Omicron Infections. N. Engl. J. Med..

[40] World Health Organization Interim Statement on Hybrid Immunity and Increasing Population Seroprevalence Rates. 1 June 2022. https://www.who.int/news/item/01-06-2022-interim-statement-on-hybrid-immunity-and-increasing-population-seroprevalence-rates.

